# Production of WW males lacking the masculine Z chromosome and mining the *Macrobrachium rosenbergii* genome for sex-chromosomes

**DOI:** 10.1038/s41598-019-47509-6

**Published:** 2019-08-27

**Authors:** Tom Levy, Ohad Rosen, Rivka Manor, Shahar Dotan, Dudu Azulay, Anna Abramov, Menachem Y. Sklarz, Vered Chalifa-Caspi, Kobi Baruch, Assaf Shechter, Amir Sagi

**Affiliations:** 1 Department of Life Sciences, Ben-Gurion University of the Negev, P.O. Box 653, Beer Sheva, 8410501 Israel; 2 Enzootic HK, Ltd., Unit 1109, 11/F, Kowloon Centre, 33 Ashley Road, Tsimshatsui, Kowloon Hong Kong; 3 The National Institute for Biotechnology in the Negev, Ben-Gurion University of the Negev, P.O. Box 653, Beer Sheva, 8410501 Israel; 4 NRGene Ltd., Ness-Ziona, 7503649 Israel

**Keywords:** Animal biotechnology, Genome

## Abstract

The cultivation of monosex populations is common in animal husbandry. However, preselecting the desired gender remains a major biotechnological and ethical challenge. To achieve an efficient biotechnology for all-female aquaculture in the economically important prawn (*Macrobrachium rosenbergii*), we achieved – for the first time – WW males using androgenic gland cells transplantation which caused full sex-reversal of WW females to functional males. Crossing the WW males with WW females yielded all-female progeny lacking the Z chromosome. We now have the ability to manipulate – by non-genomic means – all possible genotype combinations (ZZ, WZ and WW) to retain either male or female phenotypes and hence to produce monosex populations of either gender. This calls for a study of the genomic basis underlying this striking sexual plasticity, questioning the content of the W and Z chromosomes. Here, we report on the sequencing of a high-quality genome exhibiting distinguishable paternal and maternal sequences. This assembly covers ~ 87.5% of the genome and yielded a remarkable N50 value of ~ 20 × 10^6^ bp. Genomic sex markers were used to initiate the identification and validation of parts of the W and Z chromosomes for the first time in arthropods.

## Introduction

Separate cultivation of single sex populations is a common practice in animal husbandry. In many cases, specific lines are selectively bred for the advantageous traits of one gender or the other. The ability to preselect the desired gender from the cultured population presents ethical and biotechnological challenges in terms of management complications and animal welfare^1^. Nonetheless, the production of monosex populations has the potential to improve the aquaculture of many species^2,3^. In crustacean species, in particular, a significant advantage in the cultivation of monosex populations is conferred by growth rate dimorphism, leading to significantly different male and female body weights at harvest^4,5^, where the differences in body weight between the sexes may be attributed to either physiological or behavioral traits^5^. In penaeid shrimps, such as *Litopenaeus vannamei* and *Penaeus monodon*, adult females are usually larger than males^2,5–7^ and therefore all-female shrimp culture would seem advantageous. In contrast, in *Macrobrachium rosenbergii*, a freshwater prawn species characterized by three different male morphotypes of different sizes^8^, the largest specimens at harvest are found within the male population^4,9^, and, therefore, all-male aquaculture would appear to be beneficial^10–12^. Since genetic sex-determination in *M*. *rosenbergii* follows the W/Z mode^10,13–15^, all-male culture was achieved by generating ZZ ‘neo-females’ (found to be fecund) and subsequently crossing them with normal ZZ males to produce all-male progeny^10,15,16^. Indeed, several generations of prawns without the W chromosome were obtained in this way^17^.

**Figure 1:**
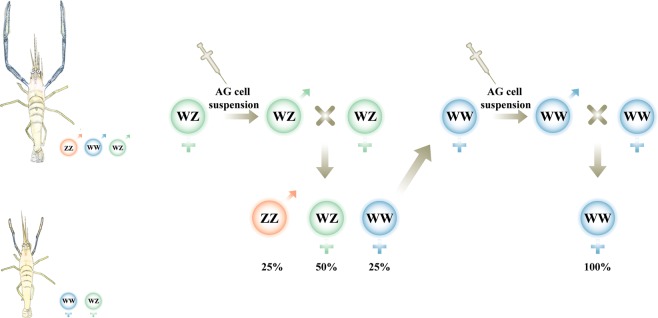
Two-phase biotechnology to produce all-female *M*. *rosenbergii* populations. (**A**) A single injection of AG cell suspension caused full sex-reversal of WZ females into WZ neo-males; the progeny of these WZ neo-males crossed with WZ females included 25% WW females. (**B**) Injection of AG cell suspension into WW females caused sex-reversal into WW neo-males; the progeny of these WW neo-males crossed with WW females yielded WW all-female populations.

The above notwithstanding, a different approach to monosex *M*. *rosenbergii* culture was suggested by Malecha *et al*.^13^, who claimed that because of the strict social structure imposed by the male morphotypes^8^, it might be more profitable to culture monosex female populations, since it is possible to stock females at higher densities due to their non-territorial and less aggressive behavior. While they did, indeed, achieve all-female progenies^13^, the complexity of their method and the low survival rate of the parental manipulated animals did not allow comprehensive testing and commercialization of their all-female vision. It was only a few decades later that a reliable, easy-to-perform and reproducible technology was established for generating all-female *M*. *rosenbergii* populations. The technology was based on transplantation of an androgenic gland (AG) cell suspension into WZ genotype females, leading to sex reversal to WZ ‘neo-males’ exhibiting the typical three male morphotypes^18^. When such neo-males were crossed with WZ females, the progeny included 25% WW females (Fig. 1A). Those WW females were found reproductively viable and were considered ‘super females,’ since crossing them with normal males (ZZ genotype) gave rise to all-female progenies^18^. With the subsequent availability of all-female mass production, the first large-scale field study testing the growth parameters of all-female versus mixed cultures was performed. Although some males in the mixed ponds were larger than most females, the field study did indeed validate the concept that the overall potential profit of all-female cultures is higher than that of mixed cultures by virtue of higher survival rates, better feed conversion ratios (FCR) and higher total crop weight. The appeal of all-female *M*. *rosenbergii* culture is strengthened even further by the more uniform body size in the all-female cultures^4^. Nonetheless, despite the substantial progress made to date, a more efficient way to produce WW super females was needed to fulfill the commercial potential of all-female *M*. *rosenbergii* cultures. To address this need, we realized that the technology now required the creation of WW neo-males (in addition to the WZ neo-males, for which the biotechnology was already available). Before we describe our efforts in this direction, a brief review of the state of the art is in place (Fig. 1).

The W/Z mode of inheritance – facilitating the production of WW super females – is not restricted to crustaceans. In avian species, the W/Z sex-determination system is well known^19^, although the production of WW females has never been reported. In fish^20,21^ and amphibians^22^, viable WW females have been produced using gynogenesis, an artificial procedure in which the sperm is deactivated prior to fertilization. In snakes, although very rare, WW super females may be born by natural virginal reproduction^23^. Similarly, in the Australian red-claw crayfish *Cherax quadricarinatus*, naturally born WW females^24^, although a very low proportion of the population^25,26^, result from crosses of females with viable intersex (WZ male) animals. Thus, while WW females do exist, there is, to the best of our knowledge, no evidence for WW males.

To the best of our knowledge, the largest region associated with sex-determination found to date in a W/Z crustacean is the linkage group (LG) 18 discovered in *L*. *vannamei*, which contains more than 90,000 markers for each sex^27^. However, functional W/Z linked sex-determining genes are yet to be identified in crustaceans. Since high-quality genome sequencing of W/Z crustacean species might increase the chances of finding such genes, some studies in this direction have been performed in the past few years in decapod species; whole genome sequencing has been performed in the cherry shrimp, *Neocaridina denticulata*^28^, the Japanese tiger prawn, *Marsupenaeus japonicus*, the giant tiger prawn, *Penaeus monodon*^29^, the marbled crayfish, *Procambarus virginalis*^30^ and recently, the Pacific white shrimp *L*. *vannamei*^31^.

In this study, we started with the production of WW neo-males, as a step in the generation of WW all-female progeny in *M*. *rosenbergii* and hence in establishing novel, elegant commercial biotechnology for all-female aquaculture. The remarkable sexual plasticity of this species – viable WW, WZ and ZZ males and females – thus gave rise to questions regarding the content of the sex-chromosomes. To initiate the mapping of these chromosomes, second and third generation techniques were used to sequence a high-quality genome of *M*. *rosenbergii*.

## Results

### Production of WW neo-males and WW progeny

**Figure 2:**
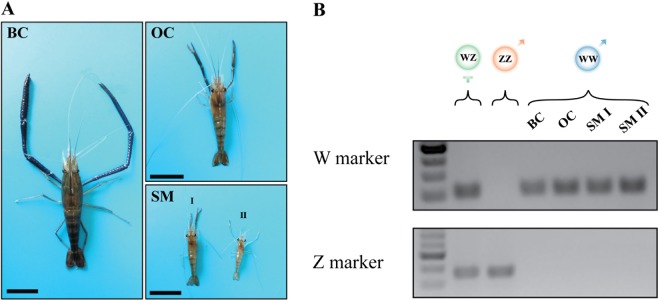
Phenotypic and genotypic characterization of *M*. *rosenbergii* WW neo-males. (**A**) Representative male morphotypes from an adult WW neo-male population: blue-claw (BC), orange-claw (OC) and two small males (SM I and II). Bars = 5 cm. (**B**) Genomic sex markers of normal female (WZ genotype), normal male (ZZ genotype) and four neo-male individuals of the three male morphotypes depicted in A (all with the WW genotype). A 100 bp DNA ladder is given.

Out of 300 post-larvae (PL) prawns that received AG transplants at age <PL_60_ and were then allowed to grow out in ponds at the Mevo Hama aquaculture facility, 259 were recovered two months post AG treatment. Examination of the male gonopores revealed 72% success of sex-reversal into phenotypic neo-males (187 animals). Eight months post injection, the neo-male population was found to exhibit the three typical *M*. *rosenbergii* male morphotypes described by Kuris *et al*.^8^, namely, blue-claw (BC) males, orange-claw (OC) males and small males (SM) (Fig. 2A), all bearing only the W chromosome, as evidenced by previously described sex-linked genomic markers^18^ (Fig. 2B).

Genomic DNA was extracted from larva samples of 24 progenies of WW females that had been crossed with the above-described WW neo-males. In addition, 20 larvae were sampled from each progeny that had been genotypically tested as described above, and all were found to bear the WW genotype. Two of the progenies, which included 46 PLs each, were retested upon metamorphosis, and all were confirmed to bear the WW genotype. In total, ~650 animals were tested, and not a single piece of evidence for the Z chromosome was found.

### Fecundity of WW females crossed with WW neo-males

Testing for fecundity by weighing the female prawn before and after hatching indicated that the mean brood somatic index (BSI) of ZZ neo-females that were fertilized by ZZ males^16^ was relatively lower than that of other females (~5% less). However, the difference was not significant (*P* = 0.07; non-parametric Kruskal-Wallis test). More importantly, the BSI of WW females that were crossed with WW neo-males was not significantly different from the BSI of WW or WZ females that were crossed with ZZ males^18^. The BSI results of the different tested groups are summarized in Table 1.

**Table 1:** Relative brood size and BSI of females bearing different genotypes.

Male	Female	n	Mean BSI (%)	SD
ZZ	ZZ	8	10.03	2.54
ZZ	WZ	11	15.33	6.53
ZZ	WW	15	16.22	12.04
WW	WW	11	15.96	4.08

The genotype of the male that was crossed with each female is indicated.

### All possible combinations of genotype/phenotype in *M*. *rosenbergii*

**Figure 3:**
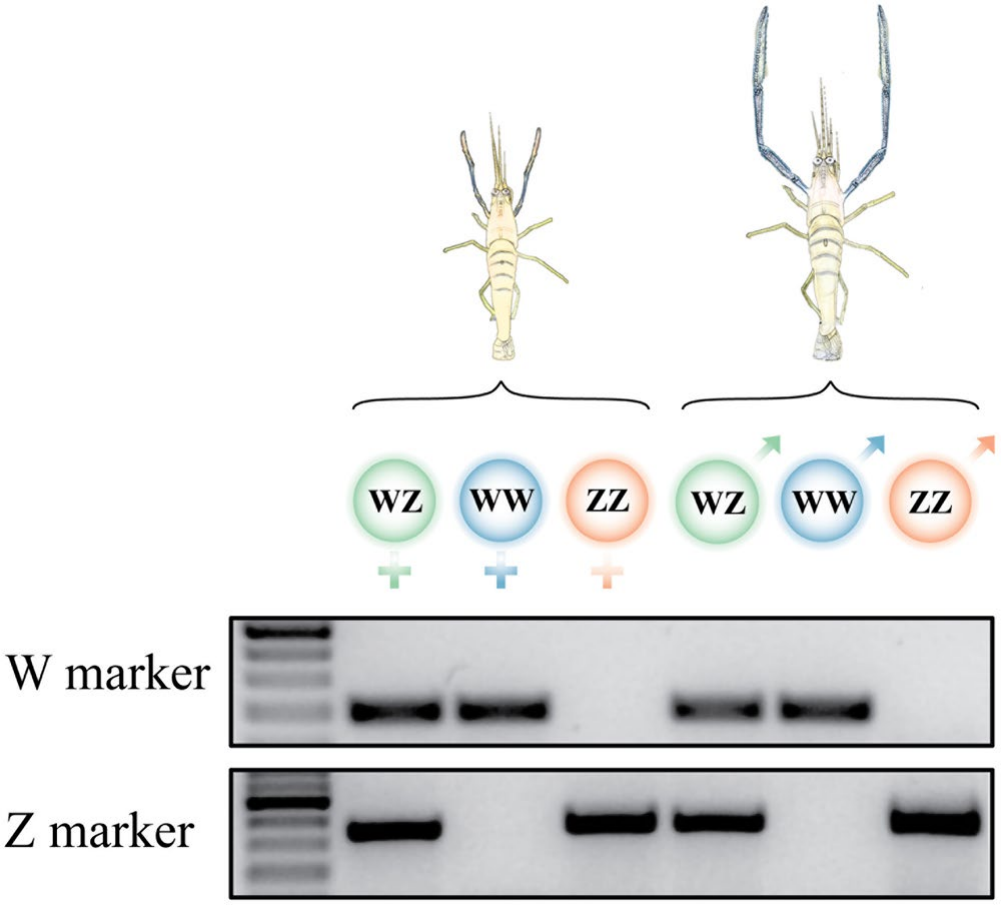
Proof, using sex specific genomic markers, of the existence of all possible genotype-phenotype combinations in *M*. *rosenbergii*. The gel showing PCR amplification of sex-specific genomic markers (W – top and Z – bottom) shows, from left to right: WZ female, WW female, ZZ female, WZ male, WW male, and ZZ male. A 100 bp DNA ladder is given.

According to our sex-specific genomic markers, all possible sex genotypes (i.e., WZ, ZZ and WW) were represented and validated in both phenotypes (i.e., males and females). A gel describing the results of the genomic testing is shown in Fig. 3.

### *M*. *rosenbergii* genome assembly and size evaluation

**Figure 4:**
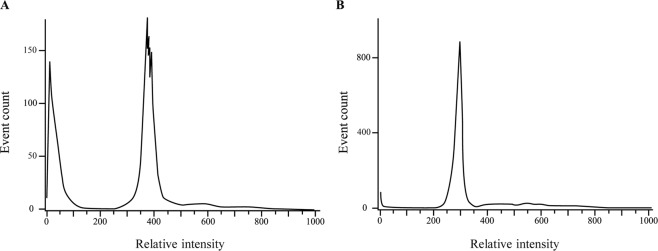
Flow cytometry for the estimation of genome size. Representative histogram of cell fluorescence relative intensity of *M*. *rosenbergii* (**A**) and *H*. *sapiens* (**B**). The Geo mean of each histogram was recorded and used to calculate the genome size of *M*. *rosenbergii* relative to the known reference, *H*. *sapiens*.

Nucleic acid staining with propidium iodide (PI) was performed to evaluate the size of the *M*. *rosenbergii* genome. Flow cytometry analysis (Fig. 4) was used to relatively quantify the genome size; the geometric mean (Geo mean) of the fluorescence relative intensity value of *M*. *rosenbergii* cells was 397.61, while that of *Homo sapiens* was 311.27. Therefore, the genome size of *M*. *rosenbergii* was calculated to be 127% of the *H*. *sapiens* genome (3.2 Gb) and estimated to be ~4.08 Gb.

DeNovoMAGIC software yielded fully assembled independent unphased and phased genomes. The unphased genome consisted of 48,584 scaffolds, with a total assembly size of 3.57 Gb and a total gap size of 306,195,586 bp, while the phased genome consisted of 1,290,365 scaffolds, with an assembly size of 6.66 Gb and a total gap size of 692,341,382 bp. In the unphased and phased assemblies, the N50 scaffold size was 19,847,992 bp and 1,705,970 bp, and the BUSCO score was 92.7% and 87.9%, respectively.

### *M*. *rosenbergii* W/Z associated scaffolds

**Figure 5:**
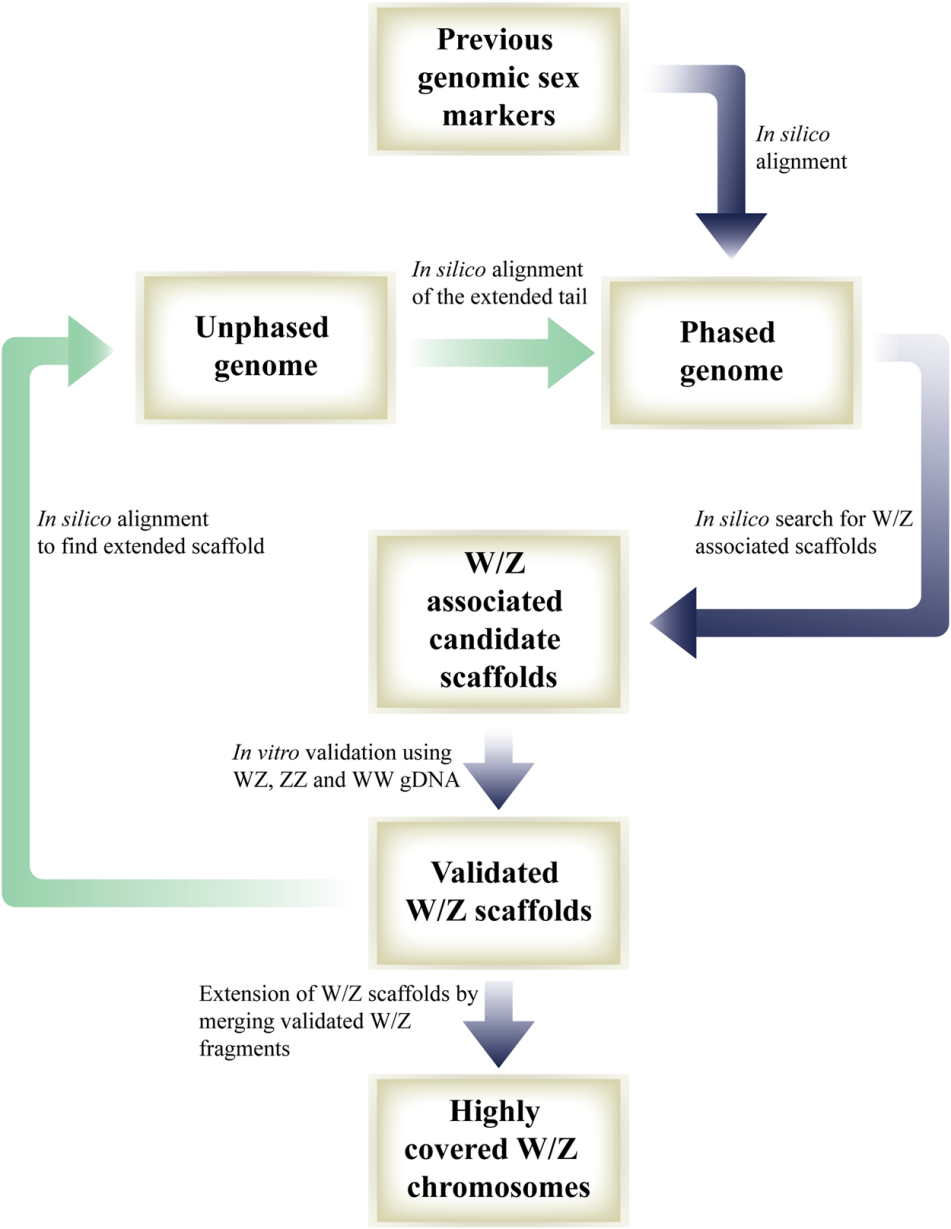
Search for W- and Z-associated scaffolds as a step towards sequencing of the sex chromosomes. Pathways to identify W- and Z-associated scaffolds (starting from our previous genomic sex markers^18^) are indicated with blue arrows, while those to extend validated W- and Z-associated scaffolds are indicated with green arrows.

As part of the process of identifying the sex chromosomes in the *M*. *rosenbergii* genome, the alignment of our previously described sex-linked genomic markers^18^ yielded an initial W-associated sequence and an initial Z-associated sequence with lengths of 5,762,597 bp and 5,109,739 bp, respectively. Applying our scaffold’s extension pipeline (Figs 5 and S1), more W/Z associated scaffolds were identified in a total length of 32,555,064 bp and 30,686,914 bp for the W and Z chromosomes, respectively. Sequence alignment of the W scaffolds with the Z scaffolds yielded 11,964 regions with potential genomic markers linked to the W chromosome and 11,094 regions with potential markers linked to the Z chromosome. Twenty-one regions specific to scaffolds associated with the W chromosome were tested using PCR and proved to be W-linked genomic markers, while five regions specific to scaffolds associated with the Z chromosome were tested and proved to be Z-linked genomic markers.

## Discussion

The novel biotechnology for the cultivation of single sex populations reported here allows us to avoid the ethical challenge of removing the unwanted gender from cultured populations. Animal welfare is thus significantly improved by the preselection of one gender—in this case all-female populations, which enable high density culture due to reduced territoriality and aggression^4^.

*M*. *rosenbergii* females normally have the WZ genotype, but homogametic ZZ^32^ and WW^18^ females have nonetheless been reported, with these homogametic females constituting the basis of commercialized biotechnologies. Male prawns normally have the ZZ genotype. We have previously reported a technology to produce heterogametic WZ males^18^, and in the current study we describe the production of homogametic WW males. Our production of homogametic WW males indicates that all possible genotype-phenotype *M*. *rosenbergii* combinations may be obtained (Fig. 3). We note that all such prawns are non-genetically modified and are now available both for research and biotechnology applications. The findings of this part of the study thus marked an important milestone towards understanding the genomic basis of the W/Z heredity system but also raised questions regarding the contents of the W and Z chromosomes. To address these questions, we conducted the genome sequencing that is discussed later on in this section.

The finding of an 87% survival rate with 72% success of phenotypic sex-reversal from female to male in female prawns injected with an AG cell suspension emphasizes the viability and the potential for commercialization of an all-female biotechnology. The potential of our novel technology is further highlighted by previous less successful attempts to produce viable neo-males by surgical procedures, which resulted in a low survival rate of ~10% with 68% success of sex-reversal^13^. The main obstacle towards the establishment of all-female prawn aquaculture was the production of WW females. In a previous study, we have reported the achievement of all-female aquaculture by three steps: sex-reversal of WZ females to WZ males, crossing the WZ males with normal WZ females to achieve a progeny that contains 25% of WW females and then crossing the WW females with normal ZZ males to achieve all-female aquaculture^18^. Upon the accomplishment of the first biotechnological phase we were able to test the viability of all-female prawn aquaculture in a large-scale field experiment^4^. Since all-female cultures were advantageous over mixed cultures^4^, a cost effective technology to produce WW females was needed. The technology, as described in this study, allows the production of WW all-female culture in one generation skipping two labor intensive steps^18^. Moreover, the molecular assays to distinguish the WW females from the progeny, which are necessary in the previous technology^18^ are no longer required as the entire progeny contains only WW females.

The presence of the known male morphotypes for *M*. *rosenbergii*^8^ in the population of WW neo-males obtained in the current study implies not only that full sex-reversal occurred but also that the male hierarchical structure was established and retained. Moreover, a particularly important aspect of this biotechnology is its reliability, as we were able to show that the entire progeny of a WW × WW cross contained only WW females. Even in terms of fecundity, BSI measurements of WW females that were crossed with either WW or ZZ males did not significantly differ from normal WZ females crossed with ZZ males^16,18^. This finding indicates that both homogametic WW females and WW males function fully – similarly to either normal males or females – and could thus serve as dams and sires for all-female cultures. While WZ and ZZ genotypes do exist naturally in this species^10,13,15,18,33^ and the natural occurrence of WW has never been reported, our current results highlight a peculiar case of sexual plasticity and hence the need for further study of the broad genomic and phenotypic implications.

We thus extended the current study to an investigation of the *M*. *rosenbergii* genome. The first step was to evaluate the genome size: when sequencing a genome for the first time, information on the estimated genome size is valuable, since it is necessary for assessing the coverage that the sequencing effort has yielded. Using flow cytometry, we empirically predicted the *M*. *rosenbergii* genome size to be ~4.08 Gb, which is within the range of genome sizes acquired by flow cytometry in most decapods (1–5 Gb; Table 2)^34–39^, but less than the previously published *M*. *rosenbergii* genome size (~6.3 Gb) that was estimated by different method^40^. The length of the *M*. *rosenbergii* unphased genome assembled in the present study (3.57 Gb) implies that 87.5% coverage of the genome was achieved. To evaluate the quality of our sequenced genome, we compared it to that of six other reported decapod genomes (Table 3)^28–30,40,41^. Although the BUSCO score was reported only for 2 out of these six genomes, we obtained a BUSCO score of 92.7%, which is, to the best of our knowledge, the highest among the reported sequenced decapod genomes. Moreover, although the coverage of the genome reported in this study is not the highest, it yielded an impressive length for the N50 value (19,847,992 bp). It is noteworthy that the N50 value obtained is ~500 times higher than its corresponding value in the highest-quality decapod genome published to date (*Procambarus virginalis*^30^). While decapod crustacean genomes are known to be highly repetitive^42^, thus causing immense difficulties in sequencing and assembling high-quality genomes, the significantly high value of N50 reported in this study is a reflection of the power of the tools that we used to assemble the *M*. *rosenbergii* genome and of the impressive quality of the genome. A distinctive feature of the DeNovoMAGIC assembler application used in this study is its ability to represent heterozygosity in the sequenced genome, and it has therefore yielded several high-quality heterozygous genome assemblies in the past^43–45^. To the best of our knowledge, the *M*. *rosenbergii* genome is the first ever arthropod genome that has been assembled as a phased genome. Together with our W and Z genomic markers^18^, this ability of the assembler to represent heterozygosity might lead us, for the first time, to deep genomic sequencing of the sex chromosomes in decapods.

**Table 2:** Reported genome sizes in decapod species that have been evaluated by flow cytometry.

Family	Species	Size [Gb]	ref.
**Palaemonidae**	***Macrobrachium rosenbergii***	**4.08**	**Present study**
Alpheidae	*Athanas nitescens*	4.73	^34^
Alvinocarididae	*Alvinocaris markensis*	10.35	^34^
Alvinocarididae	*Chorocaris chacei*	13.06	^34^
Alvinocarididae	*Mirocaris fortunata*	11.25	^34^
Alvinocarididae	*Rimicaris exoculata*	10.16	^34^
Aristaeidae	*Aristaeomorpha foliacea*	5.11	^35^
Bythograeidae	*Bythograea laubieri*	4.39	^34^
Bythograeidae	*Bythograea thermydron*	4.40	^34^
Bythograeidae	*Cyanagraea praedator*	2.97	^34^
Bythograeidae	*Segonzacia mesatlantica*	4.75	^34^
Cancridae	*Cancer pagurus*	2.39	^34^
Crangonidae	*Argis dentata*	17.04	^36^
Crangonidae	*Crangon crangon*	11.10	^34^
Crangonidae	*Crangon septemspinosa*	9.70	^36^
Crangonidae	*Sclerocrangon ferox*	39.99	^36^
Galatheidae	*Galathea squamifera*	8.27	^34^
Galatheidae	*Galathea strigosa*	6.84	^34^
Galatheidae	*Munidopsis recta*	15.22	^34^
Hippolytidae	*Bythocaris irene*	37.62	^36^
Hippolytidae	*Eualus gaimardii*	16.60	^36^
Hippolytidae	*Spirontocaris spinus*	12.93	^36^
Majidae	*Maja crispata*	3.79	^37^
Nephropidae	*Homarus americanus*	4.65	^35^
Nephropidae	*Homarus gammarus*	4.16	^35^
Nephropidae	*Nephrops norvegicus*	4.79	^35^
Palaemonidae	*Palaemon serratus*	9.99	^34^
Palinuridae	*Jasus edwardsii*	4.90	^35^
Palinuridae	*Jasus frontalis*	4.56	^35^
Palinuridae	*Jasus novaehollandiae*	5.21	^35^
Palinuridae	*Palinurus elephas*	4.18	^35^
Palinuridae	*Palinurus mauritanicus*	3.08	^35^
Pandalidae	*Pandalus montagui*	8.34	^36^
Penaeidae	*Litopenaeus vannamei*	2.45	^38^
Penaeidae	*Penaeus aztecus*	2.39	^38^
Penaeidae	*Penaeus duorarum*	2.32	^38^
Penaeidae	*Penaeus setiferus*	2.45	^38^
Porcellanidae	*Pisidia longicornis*	8.11	^34^
Porcellanidae	*Porcellana platycheles*	7.43	^34^
Portunidae	*Carcinus maenas*	1.21	^34^
Portunidae	*Charybdis japonica*	2.28	^39^
Portunidae	*Necora puber*	14.84	^34^
Portunidae	*Portunus trituberculatus*	2.26	^39^
Portunidae	*Scylla paramamosain*	1.60	^39^
Scyllaridae	*Scyllarides herklotsii*	6.67	^35^
Scyllaridae	*Scyllarides latus*	6.84	^35^
Scyllaridae	*Scyllarus arctus*	1.98	^35^
Scyllaridae	*Scyllarus pygmaeus*	1.90	^35^
Varunidae	*Eriocheir sinensis*	2.24	^39^
Xanthidae	*Xantho incisus*	4.81	^34^
Xanthidae	*Xantho pilipes*	11.51	^34^

The family and scientific name of each species are given in addition to the genome size in Gb.

**Table 3:** Genome sequencing details for decapod species.

Species	Size [Gb]	Coverage [%]	N50 [bp]	BUSCO [%]	ref.
***Macrobrachium rosenbergii***	**4.08**	**87.5**	**19,847,992**	**92.7**	**Present study**
*Exopalaemon carinicauda*	6.62	84.13	816	66	^40^
*Neocaridina denticulata*	3.00	42.67	400	—	^28^
*Penaeus monodon*	2.59	78.76	789	—	^29^
*Marsupenaeus japonicus*	2.28	85.01	937	—	^29^
*Procambarus virginalis*	3.50	94.28	39,400	88	^30^
*Litopenaeus vannamei*	2.45	96.70	1,343	—	^41^

The estimated genome size, percentage coverage, N50, and BUSCO score (if reported) are given.

Karyotypic analysis of decapod crustaceans has revealed that their chromosomes are relatively small and numerous^46^. All previous karyotyping studies in decapod crustaceans have not been successful in identifying sex chromosomes; these studies have covered a variety of decapod species, including penaeid shrimps^47^, Cambaridae crayfish^48^, Portunidae crabs^49^, and even the freshwater prawns *M*. *siwalikensis*^50^ and *M*. *rosenbergii*^51–53^. According to those studies, the *M*. *rosenbergii* genome contains 59 pairs of chromosomes. Damrongphol *et al*.^53^ classified the 59 pairs according to size, namely, large (6 pairs), medium (26 pairs), small (15 pairs) and very small (12 pairs). Based on their graphical analysis of the karyotype^53^ and our *M*. *rosenbergii* genome size estimation, it is possible that the *M*. *rosenbergii* sex-chromosome pair resides among either the 12 very small pairs, the 15 small pairs, the 26 medium pairs or the 6 large pairs (see Table S1). Further study is thus needed to precisely locate the sex-chromosomes pair among the remaining 47 chromosome pairs of *M*. *rosenbergii*. Nonetheless, even at this early stage of *M*. *rosenbergii* sex-chromosome mapping, it is clear that powerful molecular and biotechnological insights have emerged from the current genomic study, including the discovery of novel sex-specific markers and distinguishable W/Z associated regions and genomic insights into sex determination and differentiation processes.

Bringing the study of sexual plasticity in prawns to completion with the demonstration of all possible genotype-phenotype combinations within the *M*. *rosenbergii* W/Z heritability system (WW males and females, WZ males and females and ZZ males and females), together with the assembly of phased and unphased high-quality genomes, establishes *M*. *rosenbergii* as a model to study universal sex differentiation and developmental mechanisms that might be common within early evolutionary arthropods^54^. It will also open the path for the development of novel monosex biotechnologies in other cultured species.

## Methods

### Animals

*M*. *rosenbergii* BC male donors (40 ± 5 g) were reared in 600-L tanks at 28 ± 2 °C with constant aeration and a light regime of 14:10 (L:D) at the R&D facilities of Enzootic Holdings, Ltd. The prawns were fed *ad libitum* with shrimp pellets containing 30% protein. WW *M*. *rosenbergii* post-larvae (PL), obtained by cross breeding WW females with WZ neo-males, were reared in a 3.5 m^3^ U-shaped tank.

### Sex reversal of WW females into WW neo-males

The *M*. *rosenbergii* male donors were endocrinologically manipulated, leading to androgenic gland (AG) hypertrophy^18,55,56^. Ten days post manipulation, the AGs were dissected from the manipulated animals under a dissecting microscope, and the hypertrophied AG (hAG) cells were separated by enzymatic dissociation, as previously described^18^. An aliquot of cell suspension was evaluated for viability and concentration, by using Trypan blue staining and counting of the cells on a hemocytometer under a light microscope. These hAG cells were transplanted (using a micro-injector under a light microscope), in an amount of ~3 × 10^3^ hAG cells per female prawn, into the abdomens of WZ and WW females, at an age of <PL_60_ (n = 300). The injected prawns were reared in earthen ponds (each ~140 m^2^ with a water depth of 0.9 m and a water temperature of 26–28 °C) at the Mevo Hama aquaculture facility, Israel.

### Examination of masculine development

Two months post transplantation, the prawns injected with AG cells were examined for the development of male gonopores. Each animal was placed on its dorsal side, and the bases of the fifth pereiopods (walking legs) were examined for the presence or absence of male gonopores. Animals that had developed male gonopores were returned to the ponds for additional grow-out, and the other animals were removed from the ponds. Four months post injection, the prawns were examined for morphotypic differentiation, which constitutes a milestone in *M*. *rosenbergii* masculine development^8^.

### Crossing WW neo-males with WW females

Eight months post injection, the above animals were taken out of the ponds, and their genotypes were determined using specific W- and Z-linked genomic sex markers, as previously described^4,18^. WW animals with male gonopores were considered WW neo-males and were transferred to a 4-m^3^ tank together with WW females for breeding. The breeding tank was examined on a weekly basis, and each berried female was removed and placed in an individual glass tank. Upon hatching, progeny was genetically tested (using the above-mentioned sex markers) to verify that the larvae did indeed bear the WW genotype. A workflow representing the process from obtaining a WW female to the achievement of WW all-female progeny is shown in Fig. 1.

### Fecundity measurements

To assess the fecundity of the WW females that had been fertilized by WW neo-males, berried WW females (n = 11) were weighed before and after hatching of the larvae. The ratio of egg mass to body weight (BSI^16,18^) was calculated. A meta-analysis was conducted to compare these results with those previously obtained for WW females fertilized by ZZ males (n = 15), WZ females fertilized by ZZ males (n = 11)^18^, and ZZ ‘neo-females’ fertilized by ZZ males (n = 8)^16^. Since according to the Shapiro-Wilk test, the residuals of the BSI measurements were not normally distributed, differences between the measurements were tested by the non-parametric Kruskal-Wallis test using Statistica v9.0 (StatSoft, Tulsa, OK).

### Genotyping all possible phenotypes in *M*. *rosenbergii*

The second pleopods were dissected from prawns bearing different chromosome combinations: a ZZ normal male, a sex-reversed normal female (expected to be a WZ neo-male^18^), a sex-reversed super female (expected to be a WW neo-male), a WZ normal female, a super female from a WZ × WZ cross (expected to be WW^18^), and a sex-reversed neo-female that was obtained by silencing the *Mr-IAG* gene (expected to be ZZ^32^). Genomic DNA was extracted using REDExtract-N-Amp Tissue PCR Kit (Sigma, Rehovot, Israel) according to the manufacturer’s instructions, and the genotype of each animal was determined using sex-specific genomic markers for *M*. *rosenbergii*, as previously described^18^.

### Evaluating *M*. *rosenbergii* genome size

Evaluation of *M*. *rosenbergii* genome size is a meaningful step prior to *de novo* sequencing of the genome. Therefore, the haploid *M*. *rosenbergii* genome size was empirically determined by using a previously described flow cytometry protocol^57^. Briefly, hemolymph was extracted from 12 prawns and pooled. Hemocytes were retrieved from the hemolymph and stained with PI. Peripheral blood mononuclear cells (PBMCs) from *H*. *sapien*s were used as a source for a reference haploid genome with the known size of 3.2 Gb^58^. Each sample was analyzed twice, and the fluorescence relative intensity of PI in each cell was measured. In each analysis at least 10,000 events were analyzed. The Geo mean fluorescence of each cell population was calculated. The following formula was used to calculate the genome size:

$$Study\,genome\,size=\frac{(Reference\,genome\,size)\times (Study\,fluoresence\,mean)}{(Reference\,fluoresence\,mean)}$$

### gDNA extraction for second- and third-generation sequencing

*M*. *rosenbergii* high molecular weight gDNA was extracted from a WZ female by using the phenol-chloroform method as follows: 200 mg of muscle tissue was flash frozen in liquid nitrogen and then ground in a mortar and pestle. Using a clean metal spatula, the powdered tissue homogenate was transferred to a 50-ml tube preloaded with 10 ml of Proteinase K buffer (20 mg/ml) in 50 mM Tris (pH 8) and calcium acetate (1.5 mM) and incubated at 45 °C overnight. After complete digestion of the tissue, 10 ml of TE saturated phenol was added, and the sample was incubated for 1 h at room temperature. Following incubation, the lower phase was discarded using a serological pipette. The latter step was repeated twice more, and then the sample was incubated overnight (without removing the lower phase in the final repetition). Next, the lower phase was discarded, 10 ml of phenol-chloroform suspension was added to the aqueous phase, and the sample was incubated at room temperature for 1 h. Thereafter, the phenol-chloroform phase was discarded, and 100% chloroform was added, followed by a 1 h incubation at room temperature. This step was repeated twice, and after adding the chloroform for the third time, the sample was incubated overnight. Next, the lower, chloroform phase was discarded. The remaining aqueous phase, retrieved from the previous step, was supplemented with 10% v/v sodium acetate (3 M) and two volumes of ice cold ethanol (100%). The sample was mixed by gentle rotation, 3 times. Using a glass shepherd’s rod, the DNA precipitate was transferred to 30 ml of cold ethanol (70%) for 5 min. Finally, the DNA was removed from the 70% ethanol using the glass shepherd’s rod and air dried for 5 min. The dry DNA precipitate was reconstituted in 100 μL of TE buffer (0.1 M).

### Sequencing and *de novo* assembly of the *M*. *rosenbergii* genome

The *M*. *rosenbergii* gDNA samples were sequenced by NRGene (Ness-Ziona, Israel) with a second-generation sequencing technology having a total depth (coverage) of ×261 [based on an estimated total (diploid) genome size of 8.2 Gb] by using Illumina (San Diego, CA) technologies. PCR-free Pair-End (PE) and Mate-Pair (MP) libraries were used to provide accurate and precise raw data. In addition, third-generation sequencing libraries were prepared and sequenced using Illumina machines, including 10X chromium, creating additional sequencing data with a depth (coverage) of ×61. A detailed description of the sequencing data is given in Table 4. The sequencing data was processed and assembled using the DeNovoMAGIC assembler application version 3.0 (NRGene, Ness-Ziona, Israel). Contig assembly, scaffolding and gap filling were performed as previously described^43^. In addition, DeNovoMAGIC was used to assemble, independently, phased and unphased genomes. Phased genome assembly aims at assembling a heterozygous genome, in which each heterozygous region of the genome should be covered by two separate scaffolds, one of maternal origin and the other of paternal origin. The unphased genome is comprised of longer scaffolds representing the longest possible sequence per locus in the genome, and each region of the genome is covered by a single scaffold. Therefore, the N50 of the unphased assembly is expected to be higher than the N50 of the phased assembly. The integrity of the assemblies was verified with several quality-assurance procedures including the independent BUSCO benchmark^59,60^, against the “Arthropoda_odb9” database with default parameters. BUSCO is used to specifically indicate the genic region integrity, ploidy and zygosity characteristics of the assembled genome.

**Table 4:** Sequencing strategy description.

Library type	Insert size	Reads	Number of libraries produced	Approximate depth (coverage)	Total Gbp produced
PCR-free PE library (PE250 × 2)	450–470 bp	250 bp × 2	2	×67	553
PCR-free PE library (PE150 × 2)	700–800 bp	150 bp × 2	2	×57	466
MP (Nextera™ MP Gel Plus)	2–4 kbp	150 bp × 2	2	×48	391
MP (Nextera™ MP Gel Plus)	5–7 kbp	150 bp × 2	2	×45	366
MP (Nextera™ MP Gel Plus)	8–10 kbp	150 bp × 2	2	×44	360
10 × genomics™ Chromium™	N/A	150 bp × 2	2	×61	504

Summary of the sequencing data that was collected from the different types of libraries.

### Mining for W/Z-associated scaffolds

Upon sequencing and assembly of the *M*. *rosenbergii* genome, we aligned the sequence of our previously described W- and Z-associated markers^14,18^ to the phased genome. The scaffolds that matched the W- and Z-associated markers were compared using two independent approaches, as follows: (1) Using the Mauve genome aligner^61^, the scaffolds were aligned and visualized using the progressive Mauve algorithm of Mauve desktop application version 20150226. (2) Using MUMmer 3.0 genome aligner^62^, the scaffolds were compared with the nucmer script (version 3.1), and reports were created with the dnadiff script (version 1.3). “mcoords”, “qdiff” and “rdiff” reports were converted to bed format with a modified version of the script, as described in: https://sequencingforever.wordpress.com/2016/12/09/view-nucmer-alignments-in-igv/. The bed files, describing regions of similarity and dissimilarity between the scaffolds, were visualized using the Integrative Genomics Viewer (IGV)^63^.

### *In vitro* validation of the putative W and Z scaffolds

Sex-linked genomic markers derived from the putative W or Z scaffolds were tested and verified on prawn individuals bearing every possible genotype. DNA was extracted from a WZ female, a WW female, a ZZ neo-female, a WZ neo-male, a WW neo-male and a ZZ male by using PCR (94 °C for 3 min, followed by 35 cycles of 94 °C for 30 s, 57 °C for 30 s, and 72 °C for 45 s, and then by a final elongation step of 72 °C for 5 min) with the Ready Mix REDTaq kit of Sigma Aldrich (St. Louis, MO), used according to the manufacturer’s instructions. The PCR products were separated on 1.5% agarose gel.

### Extending W and Z scaffolds

To extend validated W and Z scaffolds and to obtain a higher coverage of the sex chromosomes, each scaffold from the phased assembly was realigned with the unphased assembly. Some of the scaffolds matched in the unphased assembly were longer than those in the phased assembly and had an extended ‘tail’ that was not part of the scaffold in the phased assembly. Then, the tail was aligned with the phased assembly, and a new candidate W/Z-associated scaffold was found in some cases. A scheme and illustration of the process of searching and extending the W/Z-associated scaffold are shown in Figs 5 and S1.

## Supplementary information

Supplementary Information
