# Supplementary material for: Production of WW males lacking the masculine Z chromosome and mining the Macrobrachium rosenbergii genome for sex-chromosomes
Source: Sci Rep. 2019 Aug 27;9:12408. doi: 10.1038/s41598-019-47509-6 (PMC6712010; doi:10.1038/s41598-019-47509-6)
Supplement: Supplementary file 1 — Supplementary Information [file 41598_2019_47509_MOESM1_ESM.pdf]

Production of WW males lacking the masculine Z chromosome and mining the *Macrobrachium rosenbergii* genome for sex-chromosomes

Tom Levy[1], Ohad Rosen[2], Rivka Manor[1], Shahar Dotan[2], Dudu Azulay[2] Anna Abramov[2], Menachem Y. Sklarz[3], Vered Chalifa-Caspi[3], Kobi Baruch[4], Assaf Shechter[2] and Amir Sagi[1,3*]

[1]Department of Life Sciences, Ben-Gurion University of the Negev, P.O. Box 653, Beer Sheva 84105, Israel

[2]Enzootic HK, Ltd., Unit 1109, 11/F, Kowloon Centre, 33 Ashley Road, Tsimshatsui, Kowloon, Hong Kong

[3]The National Institute for Biotechnology in the Negev, Ben-Gurion University of the Negev, P.O. Box 653, Beer Sheva 84105, Israel

[4]NRGene Ltd., Ness-Ziona, 7503649, Israel

*Author to whom correspondence should be addressed:

Email: sagia@bgu.ac.il (tel: +972-8-6461364, fax: +972-8-6479062)

Supplemental material

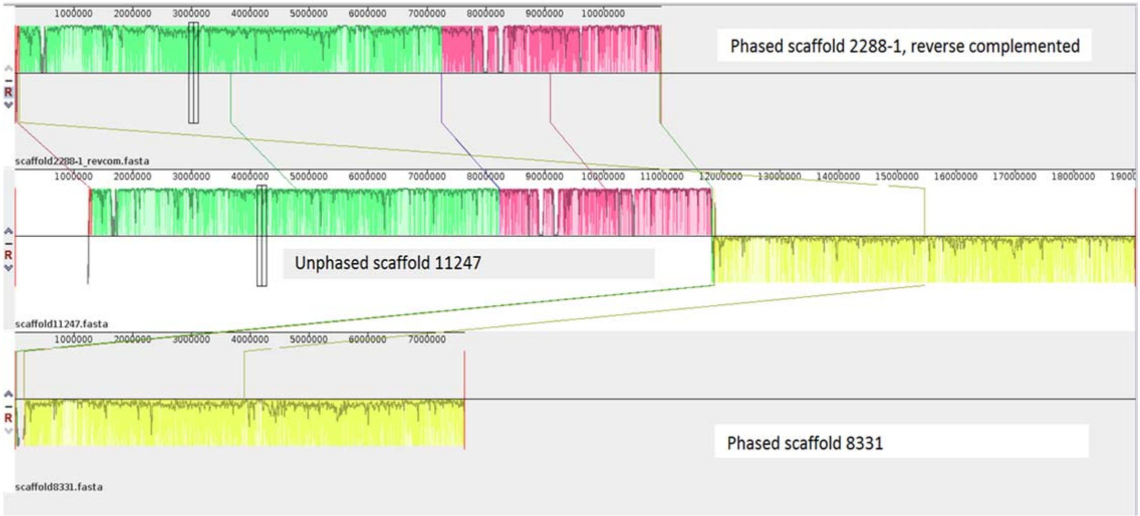

Figure S1: Representation of an extension process of W/Z-associated scaffold using the phased and unphased genomes. The process starts with a validated scaffold from the phased genome, identification of another W/Z-associated scaffold candidate via realignment to the unphased genome, and then back to the phased genome. In the following example, a verified W-associated scaffold from the phased genome (scaffold 2288-1) was matched to another scaffold (scaffold 11247) in the unphased genome, which is longer than 2288-1. Then, scaffold 11247 was realigned to the phased genome and its extension matched to another scaffold (scaffold 8331). The result is an extended W-associated scaffold candidate.

Table S1: Chromosome size prediction using our estimated haploid genome size (4.08 Gb) and the *M. rosenbergii* karyotype analysis as described by Damrongphol et al. (1991)[53].

Chromosome type	Pairs of chromosomes	Reference chromosome for calculation	Relative size per pair*	Calculated haploid size [Gb]
Very small	12	56	*X*	0.045
Small	15	34	*1.27X*	0.057
Medium	26	9	*1.68X*	0.076
Large	6	2	*2.52X*	0.113

$*Haploid\ size: 12X + (15 \times 1.27X) + (26 \times 1.68X) + (6 \times 2.52X) = 4.08\ [Gb]$

$89.85X = 4.08\ [Gb] \qquad X = 0.045\ [Gb]$

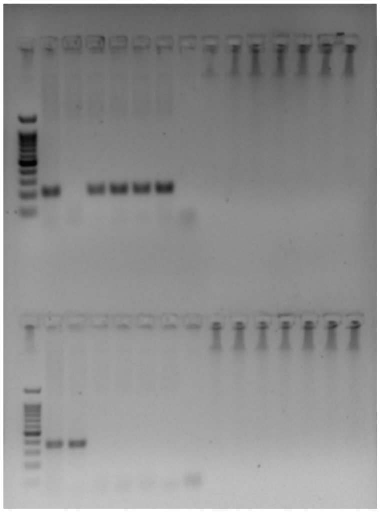

Figure S2: Genotypic characterization of *M. rosenbergii* WW neo-males (full-length gel from figure 2B). The gel showing PCR amplification of sex-specific genomic markers (W – top and Z – bottom), shows, from left to right: WZ female, ZZ male and four neo-male individuals (all with the WW genotype): BC, OC and two small males. A 100bp DNA ladder is given.

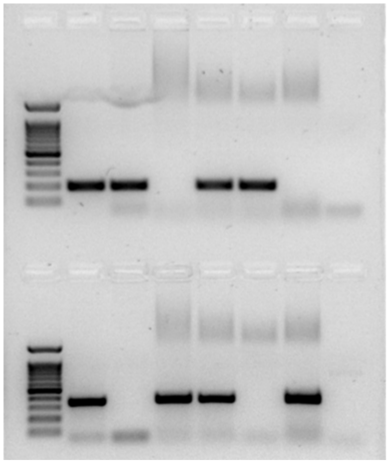

Figure S3: Proof, using sex specific genomic markers, of the existence of all possible genotype-phenotype combinations in *M. rosenbergii* (full-length gel from figure 3). The gel showing PCR amplification of sex-specific genomic markers (W – top and Z – bottom) shows, from left to right: WZ female, WW female, ZZ female, WZ male, WW male, and ZZ male. A 100bp DNA ladder is given.
